# The Development of GABAergic Network in Depression in Recent 17 Years: A Visual Analysis Based on CiteSpace and VOSviewer

**DOI:** 10.3389/fpsyt.2022.874137

**Published:** 2022-05-18

**Authors:** Jieping Lin, Fa Ling, Ping Huang, Min Chen, Min Song, Kangrong Lu, Wanshan Wang

**Affiliations:** ^1^Nanfang Hospital, Southern Medical University, Guangzhou, China; ^2^Laboratory Animal Management Center, Southern Medical University, Guangzhou, China; ^3^College of Medical Examination and Biotechnology, Southern Medical University, Guangzhou, China; ^4^Southern Medical University Library, Guangzhou, China; ^5^NMPA Key Laboratory for Safety Evaluation of Cosmetics, Southern Medical University, Guangzhou, China; ^6^Guangzhou Southern Medical Laboratory Animal Sci. and Tech. Co., Ltd., Guangzhou, China

**Keywords:** GABAergic, depression, CiteSpace, visualization analysis, VOSviewer

## Abstract

In this study, we analyzed the status and research trends of the GABAergic system in depression from 2004 to 2020 to provide a reference for further research. The Web of Science database was used as the data source and 1,658 publishments were included. Using two visualization analysis software, CiteSpace and VOSviewer, we analyzed the publishing years, countries, institutions, authors, journals, categories, keywords, and research frontiers in depression. The publishments revealed an upward trend from 2004 to 2020; the most prolific country and institutions were the United States and INSERM, respectively. The journal of Neuroscience was the most published and cited journal. The most relevant category was neurosciences. The hot topics in this field were GABAergic research in Gaba(a) receptor; the research frontier was depressive model. These analysis results provide a new perspective for researchers to conduct studies on related topics in the future and guidance for scientists to identify potential collaborators and research cooperation institutions.

## Introduction

Depression is one of the most common mental disorders, characterized by persistent depression and lack of pleasure. It includes anxiety, sleep disorders, lack of interest, hopelessness and helplessness, decreased attention, and suicidal thoughts. Worldwide, more than 350 million people are affected by depression. From 2005 to 2015, the number of patients with depression increased by 18%, making it one of the most common diseases globally (1). In 2015, the World Health Organization ranked depression as the most significant single factor for global disability, with two-thirds of suicides occurring due to depression. However, due to stigma, lack of effective treatment and insufficient mental health resources, depression is generally not diagnosed and treated (2). Depression is also difficult to treat because of phenotypic diversity and etiological heterogeneity (3). Depression is caused by several factors, including genetic and environmental factors. The underlying neurobiological determinants of depression remain unclear despite the efforts in exploring its pathophysiological mechanisms. First- and second-generation antidepressants are mainly based on the monoamine hypothesis. First-generation antidepressants include monoamine oxidase inhibitor (MAOI) and tricyclic drug (TCA), which inhibit the oxidation of monoamine and increase the extracellular concentration of 5-hydroxytryptamine (5-HT), dopamine (DA), and noradrenaline (NE) (4–6). Second-generation antidepressants, such as selective serotonin reuptake inhibitors (SSRIs), can increase 5-HT concentrations in the whole brain. At present, monoaminergic drugs, especially SSRIs, are recommended as the first-line therapy at home and abroad. However, monoamine drugs take weeks and months to produce a treatment response, and one-third of patients develop treatment resistance (7).

Considering the relatively low efficacy of monoamine drugs, new drugs with higher efficacy must be developed to resolve the current treatment limitations. Studies have reported the role of the GABAergic system in the pathogenesis of depression, antidepressants, anxiety, and schizophrenia. In 2011, Luscher proposed the GABAergic deficit hypothesis (8), which indicated an association among depressive symptoms, the gamma-aminobutyric acid (GABA) system, and GABA receptor deficiency (9). Stress regulation is based on GABAergic transmission, and chronic stress is the most critical vulnerability factor of major depressive disorder (MDD) (10). Mental pressure can also result in the loss of GABA function, caused by the change of reversal potential due to a decrease in the transmembrane KCl anion cotransporter function (11). However, so far, different neuropsychiatric diseases could not be distinguished by mutations or functional polymorphisms of genes closely related to GABAergic transmission, which must be further explored.

GABA is a crucial inhibitory neurotransmitter of the central nervous system (CNS). GABAergic neurons are located in the hippocampus (HPC), thalamus, basal ganglia, hypothalamus, and brainstem. GABA is synthesized by glutamate decarboxylases (GAD65 and GAD67) in the cytoplasm of presynaptic neurons. After synthesis, GABA is loaded into synaptic vesicles through vesicular inhibitory amino acid transporters (VGAT1 and VGAT2). The SNARE (soluble N-ethylmaleimide-sensitive factor attachment protein receptor) complex helps to couple the vesicles to the plasma membrane of the cell. When the action potential reaches the presynaptic cell, the voltage-gated calcium channel opens, and calcium binds to a synaptic binding protein, fusing vesicles and plasma membrane and releasing GABA into the synaptic gap bind with the GABA receptor. GABA can then be degraded or transported back to glial or presynaptic cells. GABA is degraded into succinic semialdehyde by GABA transaminase and enters the citric acid cycle (12, 13).

Early pioneering studies have revealed that patients with depression have lower plasma and cerebrospinal fluid GABA levels (14–16). The introduction of magnetic resonance spectroscopy (MRS) can directly and non-invasively measure GABA levels in the brain. Studies have indicated that GABA levels in the prefrontal cortex (PFC), anterior cingulate cortex, and occipital lobe are decreased in patients with MDD (17–20). This is the most substantial evidence that GABA deficiency may cause depression. Gabbay et al. concluded that the therapeutic effect of SSRIs may involve a GABAergic mechanism (21). Additionally, SSRIs increase GABA levels in the brain by stimulating the 5-HT2B receptor of astrocytes (22). Transcranial magnetic stimulation (TMS) can regulate cortical GABAergic and glutamatergic imbalance. TMS has been widely used in adult clinical treatment and is highly considered an experimental treatment for depression in adolescents who do not respond to conventional treatments, such as cognitive behavioral therapy and SSRIs. However, unknown factors related to neurodevelopment and TMS exposure in adolescents must be considered (23).

With the development of research, the GABAergic system plays an increasingly crucial role in depression. In this study, we discussed the research status, hot spots, and development trend of the GABAergic system in the field of depression through the visual analysis of literature data by CiteSpace to provide a reference for research in related areas in the future.

## Materials and Methods

### Data Acquisition

**Figure 1:**
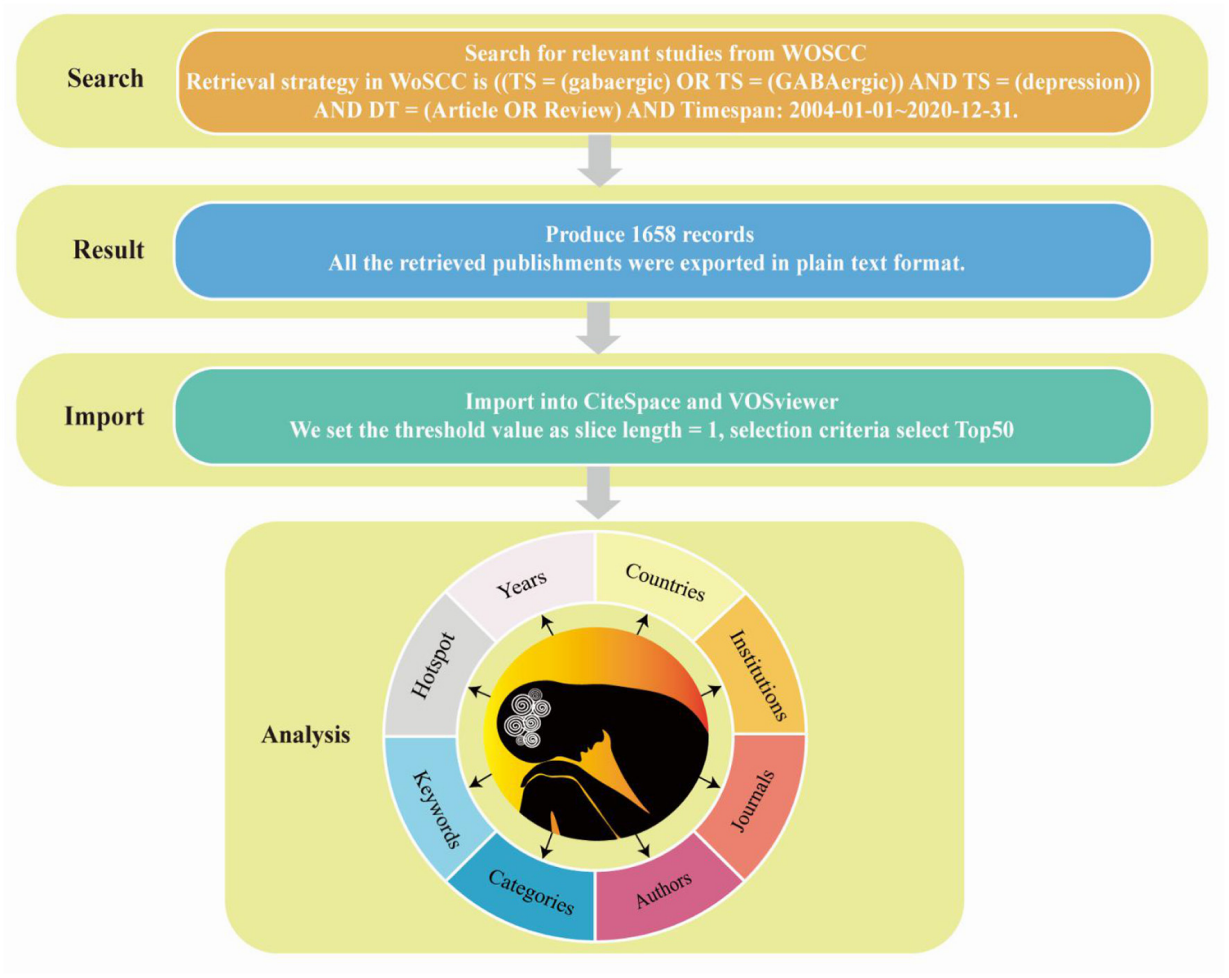
Flow chart of retrieving publishments from Web of Science Core Collection, obtaining publishments and importing them into CiteSpace and VOSviewer for analysis.

Data for GABAergic and depression analysis were collected from the Web of Science Core Collection (WoSCC), including SCI-Expanded, one of the most influential scientific literature databases, on 1 August 2021. The retrieval strategy in WoSCC is [(TS = (gabaergic) OR TS = (GABAergic)] AND TS = (depression) AND DT = (Article OR Review) AND Timespan: 2004-01-01–2020-12-31. The search produced 1,658 records, and only the original articles and reviews were included (Figure 1). All the retrieved publishments were exported in plain text format.

### Statistical Analysis

Origin is a scientific drawing and data analysis software developed by the OriginLab company. In the current study, we drew charts using Origing 2018 to display trends between publishments and year intuitively.

CiteSpace is a visualization software for creating scientific knowledge maps. CiteSpace draws the knowledge map of related fields, directly displays the information panorama of a certain knowledge field, identifies imperative literature, hot research, and Frontier direction of a certain scientific field through diversified and dynamic network analysis (24). Node centrality is an index to quantify the importance of nodes in the network; higher centrality indicates more contacts in the network that must pass through the node, indicating its vital role in communicating with other nodes and acting as a bridge. As the representative of core words with high frequency in papers, a keyword embodies crucial information. The module value (Q value) and average contour value (S value) are calculated according to the network structure and clustering clarity in the keyword clustering network. Q > 0.3 indicates significant clustering structure, S > 0.5 indicates reasonable clustering, and S > 0.7 indicates reliable clustering. In addition, the variant words of a topic in the future are a sign of the sudden growth of hot spots in the field and a valuable measure of the future development trend of the topic. The mutation value represents the mutation size; the larger the variation value, the more obvious the developmental trend of topics related to variation words. Using the bibliometric research method, we used the CiteSpace 5.8.R3 software to discuss the years, countries, institutions, journals, categories, keywords and hotspot from 2004 to 2020 (Figure 1). We set the threshold value as slice length = 1, selection criteria select Top50, respectively select “country,” “institution,” “keyword,” “category,” four nodes to explore the author, country, institution and keywords of publishments, and selected “cited author,” and “cited journal” three nodes to explore the co-citation of papers.

VOSviewer is a bibliometric analysis software, which is easy to operate and presents a more concise diagram than CiteSpace. The results were shown in two forms, namely, general view and thermal diagram. This paper used it to analyse countries, institutions and keywords.

## Results

### Research Trends of the GABAergic System and Depression

**Figure 2:**
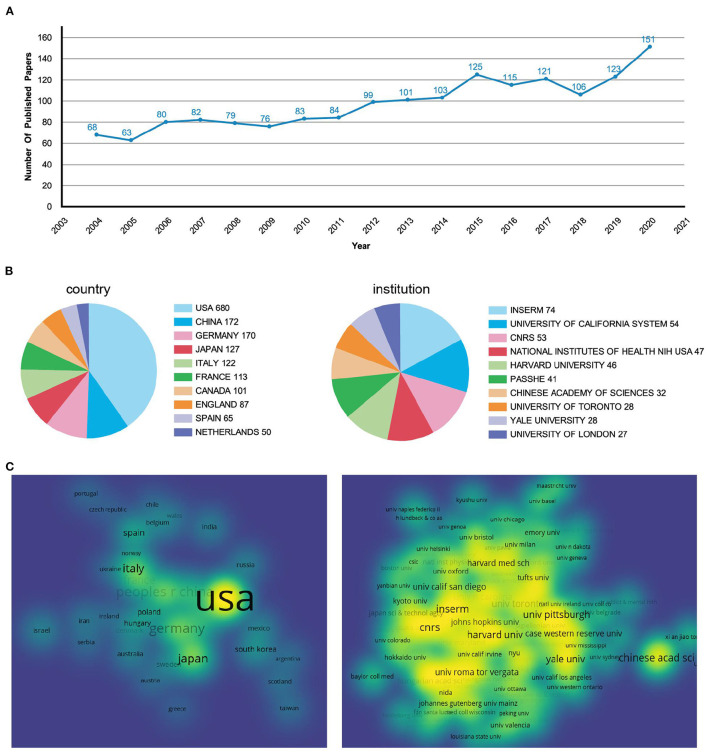
Analysis of the annual publishments, countries, and institutions. **(A)** Line graphs of papers published every year from 2004 to 2020. **(B)** Pie chart of publishment issued by countries and institutions. **(C)** The density visualization of countries and institutions.

The number and changes of published papers in a certain period are important indicators to measure the research status in related fields. Figure 2A showed the number of publishments related to GABAergic system and depression over the years. From 2004 to 2020, the overall number of publishments showed an upward trend, indicating that the research had attracted more attention. Before 2015, the number of publishments increased year by year. From 2016 to 2018, the number of publishments decreased and fluctuated slightly compared with 2015. In the past 3 years, the publishing volume had increased rapidly. By 2020, the number of publishments had doubled from 2004.

### Visual Analysis of Countries and Institutions

Among the papers published in the research field of depression and GABAergic system, the United States had the largest number, with 680 (41%), which was much higher than that of other countries (Figure 2B). China, Germany, Japan and Italy ranked second to fifth. In the visual density map, the area and brightness of the United States were also much higher than those of other countries (Figure 2C). The visual density map was made by VOSviewer. In Figure 2C, if the density of countries and institutions was smaller, the color of nodes was closer to blue; if the density of countries or institutions was bigger, the color of nodes was closer to yellow, indicating the closer relationship between countries or institutions.

A total of 1,469 institutions participated in the issuance of papers, of which 6 institutions issued more than 2%. The institution with the highest number of articles was Institut National de la Sant é et de la recherche médicale (INSERM), which had published 74 articles (4.5%) (Figure 2B). INSERM, founded in 1964, is the only public research organization fully committed to human health in France (https://www.inserm.fr/). Although France was not among the top five countries with the largest number of publishments, the institution with the largest number of publishments was in France. In the visual density map, its color was yellow and its area was large, indicating that it had carried out close cooperation with the world's top research institutions in the past 17 years (Figure 2C).

### Analysis of Co-authors

**Figure 3:**
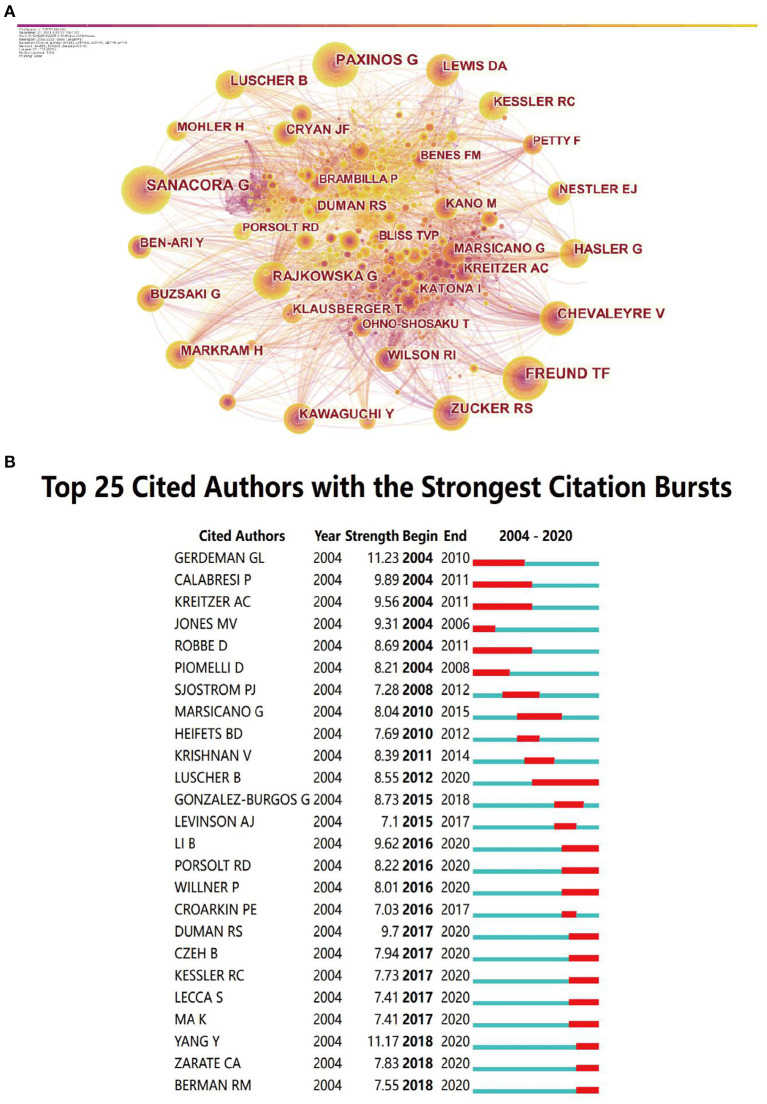
The analysis of cited-authors. **(A)** Visualization of cited authors. **(B)** The top cited authors with the strongest citation bursts.

Through the co-analysis of authors by CiteSpace, we can show the core authors in a discipline or research field and their cooperation intensity and mutual citation relationship. From 2004 to 2020, the map (Figure 3A) had 866 nodes and 5,249 connections, and the network density was 0.014. It can be seen from the map that Sanacora G, Paxinos G and Freund TF appeared the most frequently, indicating that their papers was cited more and played an important role in this field.

Among the strongest citation bursts of the top 25 cited authors from 2004 to 2005, Luscher was the author with the longest duration, from 2012 to 2020, indicating that his articles had a great impact after publishment (Figure 3B). The red bars indicated keywords cited frequently; the green bars indicated keywords cited infrequently.

### Analysis of Journals and Cited Journals

**Figure 4:**
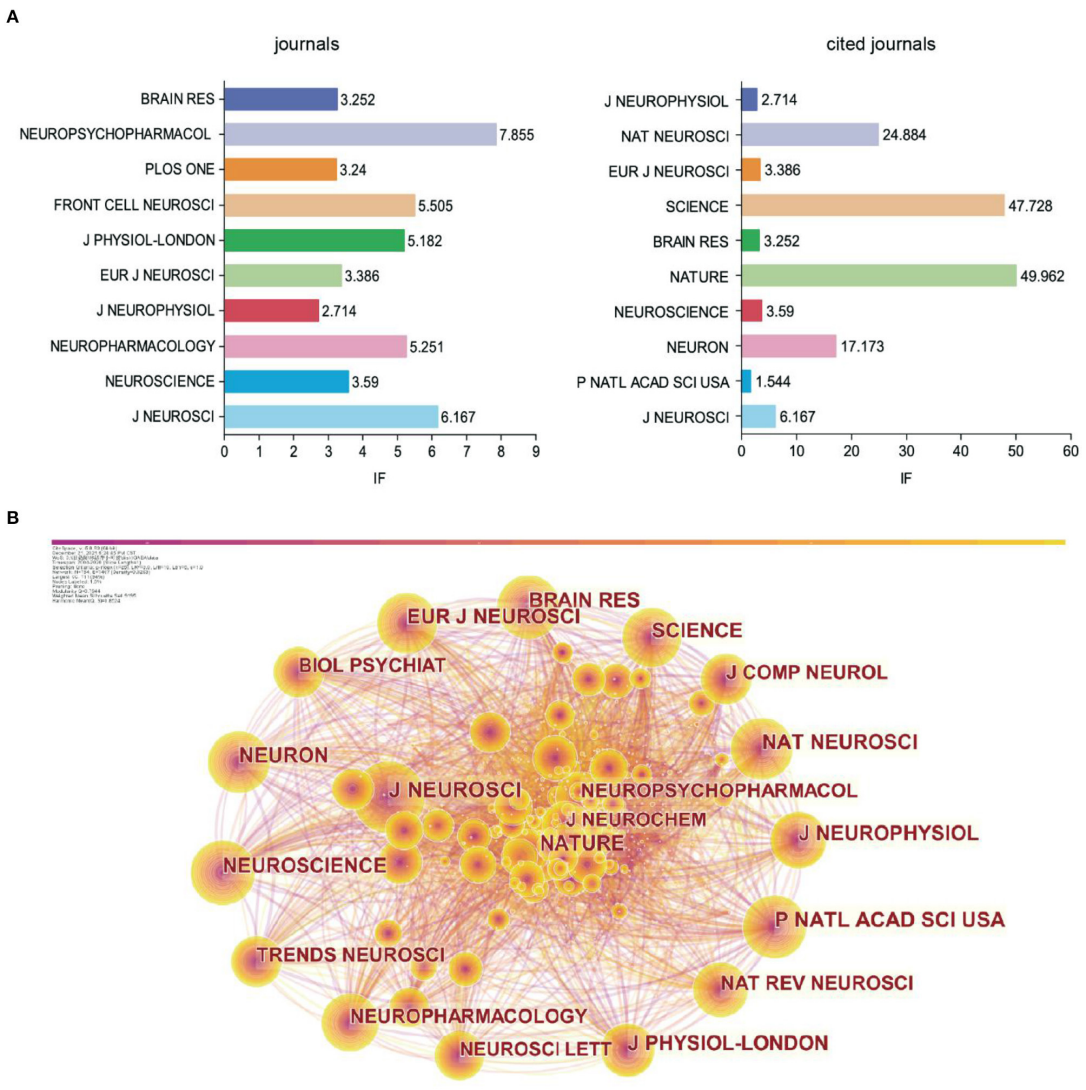
The analysis of cited-journal. **(A)** Bar chart of journals and cited journals and its IF. **(B)** Visualization of cited journals.

The 1,659 publishments involved 97 journals. The top 10 journals with published articles and the top 10 journals with cited times were shown in Figure 4A. The Journal of Neuroscience was ranked first, followed by Neuroscience, Neuropharmacology, Journal of Neurophysiology, and European Journal of Neuroscience. For all the top 10 journals with published articles, the impact factor (IF) was <10. The IF was acquired from Clarivate Analytics' Journal Citation Reports.

The IF of co-cited journals was generally higher than that of published journals. In addition, the Journal of Neuroscience ranked first among the co-cited journals. The Journal of Neuroscience had the largest node in the visual map (Figure 4B). Combining the findings presented in Figure 4A, we confirmed that the Journal of Neuroscience was the core journal with the most relevant papers, indicating its comprehensive strength and influence over the other journals in GABAergic research of depression.

### Analysis of Categories

**Figure 5:**
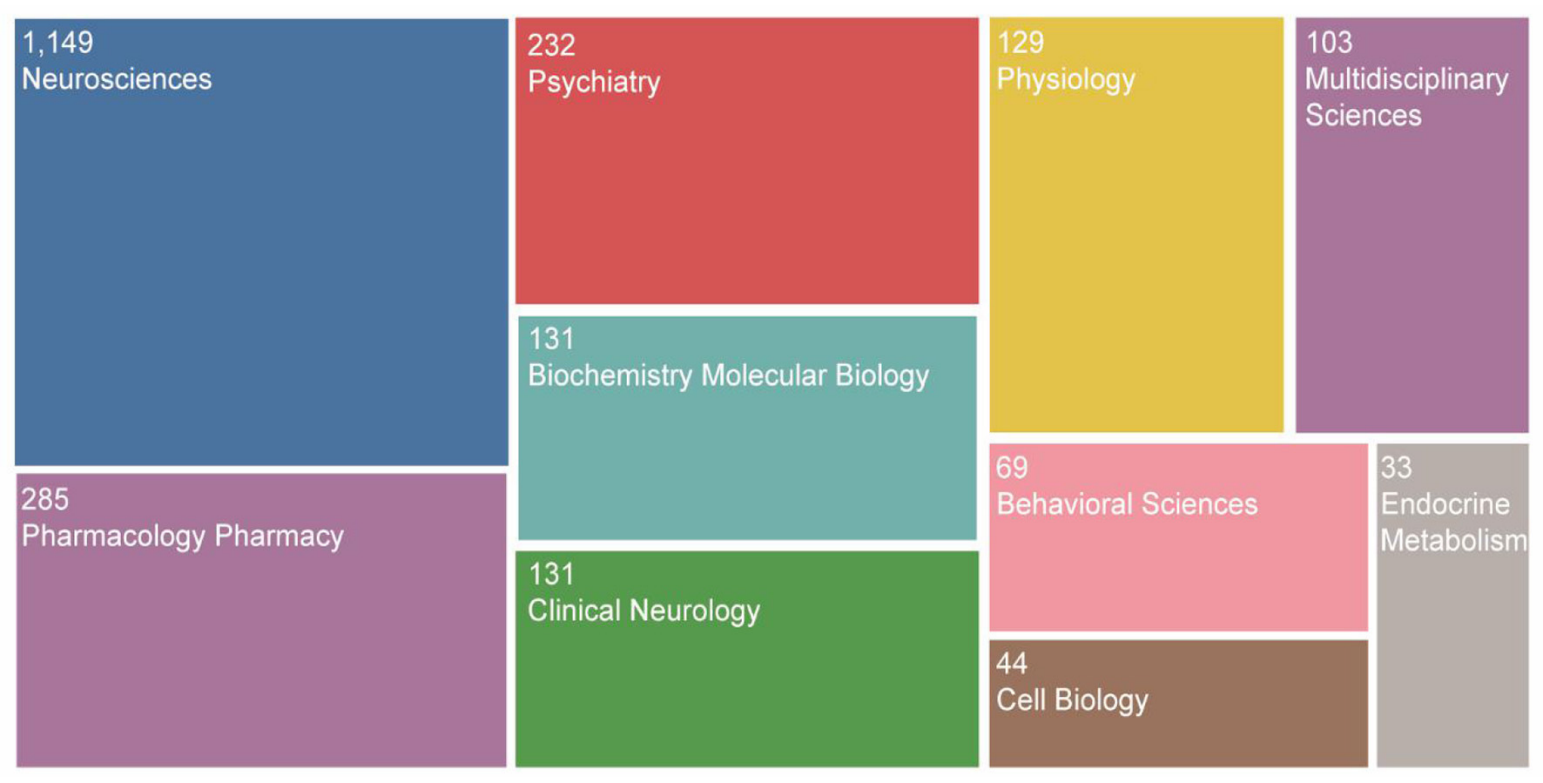
The top 10 categories.

GABAergic research in depression primarily involved the field of Neuroscience and Pharmacology, Psychiatry, Biochemistry Molecular Biology and Clinical Neurology. The top 10 categories were shown in Figure 5. In addition, it also involved the multidisciplinary science and endocrine metabolism of depression, indicating that its research and application in these two directions had further exploration value.

### Analysis of Keywords

**Figure 6:**
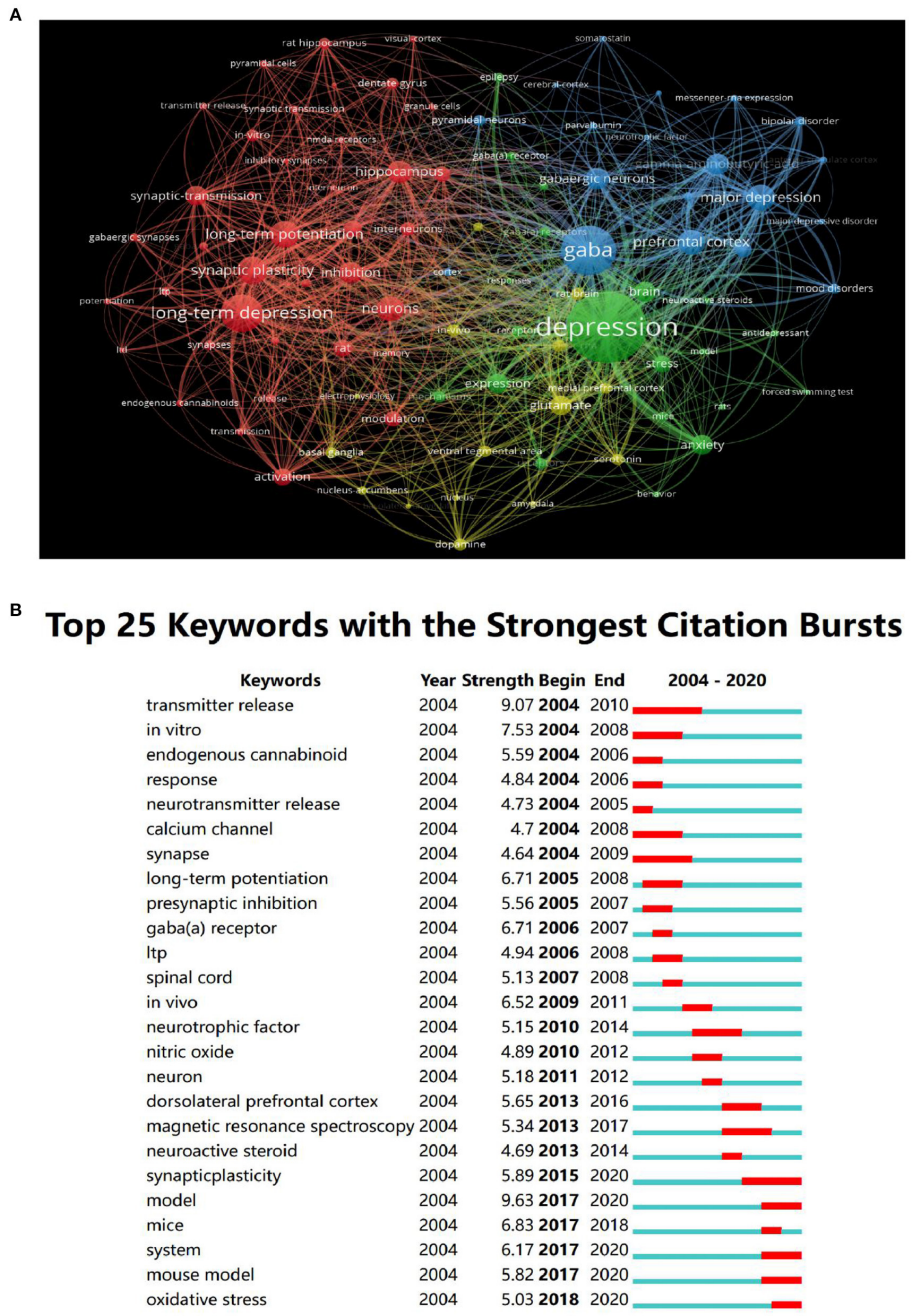
The analysis of keywords. **(A)** Visualization of keywords. **(B)** The top 25 keywords with the strongest citation bursts.

Statistics revealed 491 keywords in the papers related to the GABAergic system of depression from 2004 to 2020. As shown in Table 1, the top 10 keywords with the highest centrality ranking were more than 0.05, but only one keyword had strong centrality (>0.1). The crucial central node was the “GABA(a) receptor,” indicating that the research of GABAergic system in depression mainly focuses on GABA(a) receptor. The primary keywords in the literature related to the study of the GABAergic system of visual depression were shown in Figure 6A. The node size indicated the frequency of referencing keywords; the higher the frequency, the larger the corresponding node. The visualization map divided the keywords into three categories: red for related mechanisms, blue for drug research, and green for clinical research.

**Table 1:** The top 10 centrality of keyword.

**Rank**	**Keyword**	**Centrality**	**Number**
1	Gaba(a) receptor	0.10	103
2	Hippocampus	0.07	135
3	Synapse	0.07	77
4	Central nervous system	0.07	57
5	Cerebral cortex	0.07	35
6	Rat	0.06	142
7	Receptor	0.06	127
8	GABAergic neuron	0.06	104
9	Serotonin	0.06	62
10	Dentate gyrus	0.06	59

At present, the rodent model of depression is more mature, and relevant research is also more extensive. The application of the depression model in non-human primates, which are closely related to human beings, is still under exploration, and primates have a better simulation effect than rodents (25). Some chronic stress models model depressive behaviors in rodents, such as chronic restraint stress, chronic unpredictable stress (CUS), and chronic social frustration stress (CSDS) (26). Chronic unpredictable mild stress (CUMS) is a widely accepted model to induce depression-like behavior in rodents (27). In the brain, chronic stress changes the function of neurotransmitters and appropriate neuroplasticity, resulting in human depression (28). Therefore, this is widely used by researchers as a depression model in rodents. The CUMS model summarizes several core behavioral characteristics of human depression. In addition to depression, the cumulative effect of CUMS may result in transient overload *in vivo*, and it may lead to systemic diseases, such as atrophy of neurons in the HPC and PFC, myocardial ischaemia, abnormal liver metabolism, and poor renal prognosis (29). Astrocytes are a central target for identifying newer and more effective treatments for depression.

### Hot Research Analysis

Figure 6B showed the top 10 keywords, of which the keyword with the most robust citation bursts was “transmitter release,” and the duration was from 2004 to 2010. The current research Frontier trends were “synaptic plasticity,” “MRS,” “pyramidal neurons.”

Synaptic plasticity was the research Frontier in this field. Decreased synaptic plasticity and altered excitatory or inhibitory balance were considered potential mechanisms of depression to study the effect of GABA on synaptic plasticity, especially in the PFC (30). The role of astrocytes in regulating neuronal activity and plasticity suggests that astrocytes are the central target of new and more effective treatments for depression. In addition, it also proves the importance of developing future treatment strategies using cell type specific drug delivery. In addition, NMDA receptor (NMDAR) was a unique ionic glutamate receptor that played a vital role in neural plasticity. The decline of plasticity caused by the deterioration of the NMDAR function results in learning and memory impairment. Learning and memory dysfunction in severe depression was related to the deterioration of the NMDAR function. Blocking NMDAR in a depression-like state can lead to improvement or remission of symptoms (31).

## Discussion

### Summary of Findings

Using CiteSpace and VOSviewer, we analyzed the publishing years, countries, institutions, authors, journals, categories, keywords, and research frontiers in depression. The publishments revealed an upward trend from 2004 to 2020; the most prolific country and institutions were the United States and INSERM, respectively. The Journal of Neuroscience was the most published and cited journal. The most relevant category was neurosciences. The hot topics in this field were GABAergic research in Gaba(a) receptor; the research frontier was depressive model.

There were two reasons for the rapid growth in the publishments on GABA in depression in the past 3 years. First, it was inseparable from the policies of various countries. According to the survey statistics of the World Health Organization, from 2017 to 2020, the proportion of countries in which the treatment of patients with specific mental health conditions (psychosis, bipolar disorder and depression) was included in the national health insurance or reimbursement plan increased from 73% in 2017 to 80% in 2020 (32).

Another major reason affecting the publishment volume was that the outbreak of coronavirus at the end of 2019 and the epidemic by 2020 had affected individual mental health, including patients, individuals contacting patients and medical personnel, and worsened many determinants of mental health through direct psychological effects and long-term economic and social consequences (33–35). In the first year of the COVID-19 pandemic, global prevalence of anxiety and depression increased by a massive 25%, according to a scientific brief released by the World Health Organization (WHO) (36). A systematic review was published in the Lancet, collecting data on mental illness patients from various countries and regions in the world from January 2020 to January 2021 and compared the prevalence of depression and anxiety before and after COVID-19, which showed that the incidence rate of psychological diseases increased significantly during COVID-19 period (37).

### Pathogenesis of GABAergic-Related Depression

There is increasing evidence that GABAergic system changes in depression. In 1980, clinical statistics showed that the GABA level in lumbar cerebrospinal fluid of patients with depression decreased, which was proved after the appearance of nuclear magnetic resonance spectroscopy (18, 38). GABA levels in plasma or cerebrospinal fluid are normal in other major mental diseases, such as schizophrenia and anxiety (39). The density of GAD65 or GAD67-immunoreactivity in hypothalamic paraventricular nucleus of patients with depression decreased, which was significant in major depression (40). Somatostatin (SST) is a γ-Neuropeptides expressed in GABA interneuron subtypes target the dendrites of pyramidal neurons. It was found that the density of SST-labeled neurons in lateral amygdala, lateral basolateral nucleus and medial basolateral nucleus decreased significantly in patients with MDD (41). The functional defect of α1βγ2-adrenoceptor may increase the GABA release of the ventral tegmental area interneurons and enhance the GABAergic inhibition of the adjacent dopaminergic neurons, inducing loss of pleasure, the core symptom of severe depressive disorder (8, 42).

Chronic stress is one of the inducing factors of depression. In rodents, stress-induced behavioral changes were related to decreased glutamate decarboxylase expression, vesicles and plasma membrane transporters during GABA release, injury of GABAergic interneurons, and reduced density and function GABAergic synapses in addition (43–45). Western blot analysis and quantitative real-time PCR have shown that CUS exposure for 5 weeks could significantly reduce the GAD67 protein levels in the PFC of rats (46). Chronic stress can also change chloride ion reversal potential to depolarisation membrane potential (47). Benzodiazepines can enhance GABA activity, thereby opening chloride channels and allowing chloride ions to enter neurons (27). However, the long-term use of benzodiazepine results in a decrease in GABA levels; the resulting depolarization of neurons blocks the action potential and induces anti-anxiety, muscle relaxing, sedative, and antiepileptic activities (48).

### GABA Receptor

GABA receptors are divided into GABA-A receptor (GABAAR) and GABA-B receptor (GABABR) subtypes (49). GABA-C receptor is usually classified as a subtype of the GABAAR, named GABA-A-rho (50). The GABAAR is a ligand-gated chloride channel (ionic type). It is a tetramer or pentamer uniquely composed of multiple subunits. At present, at least 19 different GABAAR subunits (termed α1-6, β1-3, γ1-3, δ, ε, π, θ and ρ1-3) have been reported (51).

GABAAR mediates rapid synaptic transmission, which is located in the postsynaptic membrane. The level of GABAAR and its subnunites changes in some patients with depression. Fatemi et al. revealed that postnatal lipopolysaccharide exposure can damage adult hippocampal neurogenesis and trigger the down-regulation of GABAAR in the later stage through early astrocyte activation, leading to depression like behavior (52). The GABRα1 subunit is expressed in most GABAARs. Some studies has found that GABRα1 protein level was significantly increased in lateral cerebellum of subjects with major depression (53). Therefore, drugs targeting GABAAR sites have also been used to treat depression. In the 1940s, Selye proved that some pregnane steroids have the effect of rapid sedation and anesthesia (54). Furthermore, electrophysiological experiments of rat brains that general steroid anesthesia enhances the stimulation of exogenous GABAAR (55). GABA receptor subunits are assembled near the central chloride pore and have been widely described as the target of several psychotropic drugs, such as the GABA-A agonist/antagonist site. Benzodiazepines bind to the GABAAR and increase the permeability of chloride ions by changing the opening frequency of the chloride channel (56). α2/α3 GABAAR modulators, such as TPA023, are a new type of anti-anxiety drugs that are superior to traditional benzodiazepines because they lack sedative effect and drug dependency (57).

GABABR is a G protein-coupled receptor, a heterodimer composed of GABAB1 and GABAB2 subunits; they are mainly located in the presynaptic region act as their receptors to inhibit the release of GABA (40). GABA-B mediates slow synaptic transmission related to memory, emotion, and pain (58, 59). The activation of GABABR can inhibit the transmission of serotonergic, and the decrease of serotonergic neuron activity is related to the development of depression (60, 61).

### GABAergic System in the Treatment of Depression

At present, the first-line treatment of depression includes antidepressants and cognitive behavioral therapy, but 30–50% of patients are ineffective (62). Studies have shown that after successful maintenance treatment of acute TMS can sometimes alleviate for 3 months or even 8 years (63–65). In refractory depression, TMS reduced the inhibitory neurophysiological markers of GABA receptor-mediated depression (N45 and N100) (66, 67). Scangos et al. (68) improved patients' depression rapidly and continuously through closed-loop therapy. Other treatments include electroconvulsive therapy, transcranial direct current stimulation and vagus nerve stimulation (69–73). Traditional antidepressants mainly inhibit monoamine transporters. Monoamine antidepressants have excellent limitations, such as low efficacy, delayed treatment, and most importantly, treatment unresponsiveness in some patients (estimated to be one-third of patients with depression) (74–76). Practicing yoga at least once a week helps maintain the improvement of GABA levels (77).

The drug treatment of depression needs to improve and go beyond the limitations of monoamine system, and may even put forward new ideas to improve the treatment effect of patients. A diminished GABAergic input to the hypothalamic paraventricular nucleus may contribute to the activation of corticotropin releasing hormone-immunoreactivity neurons in depression, most prominently in major depression, which provides a rationale for prescribing GABAergic agonists for these patients (40). The emergence of new rapid drugs for the GABA system has solved specific problems and provided better therapeutic intervention for this persistent disease (78). The emergence of ketamine, a new rapid drug for GABA system, solves specific problems and provides better therapeutic intervention for this persistent disease (78). The initial cellular trigger of ketamine's rapid antidepressant effect is Glun2b NMDAR on GABA interneurons (16). Ketamine has recently been approved for the treatment of refractory depression, and its dose and plasma concentration are positively correlated with antidepressant response (79, 80).

Enhancing the transmission of GABA is the core of some new antidepressant treatments. Several studies have supported that primary excitatory neuron play a role in the GABAergic neuroactive steroids (NASS) synthesis, an endogenous steroid synthesized by cholesterol in the brain and nervous system (81–83). NASS can regulate the surface expression of the GABAAR. However, unlike phenylene bisulfide, they only regulate the receptor containing gamma subunit; based on the multichannel characteristics of the GABAAR, positive allosteric modulators may improve the therapeutic effect (84, 85). This suggests that a novel technique to treat depression is to develop novel antidepressants that can enhance the function of GABA and act on GABA receptors. The deletion of the GABA receptor-γ2 subunit increases GABA inhibition and results in anti-anxiety and antidepressant behaviors (45). Cognitive impairment is now considered the core symptom of depression and other mental disorders (86–88). A decrease in the signal pathway of SST + neurons/α5-GABA receptor pathway will lead to cognitive dysfunction, representing a new treatment target for treating cognitive disorder symptoms of depression (45). However, more studies must be carried out on the association of GABAergic deficiency with other pathophysiological changes of depression, such as inflammation, apoptosis, and oligodendrocyte dysfunction (89–91).

## Conclusion

Based on the CiteSpace, VOSviewer, and WoSCC, the current study performed a bibliometric analysis and in-depth interpretation on the research of the GABAergic system in depression in the last 10 years from the aspects of the number of studies published, co-occurrence of research authors, journals, countries, categories, institutions, keywords, and research hotspots. Our study's findings revealed that the research had developed rapidly over the past 17 years, especially in the last 5 years. Further developments in GABAergic research are promoting the exploration of the pathogenesis of depression to a certain degree. In this review, we systematically summarized the basic information and pathogenesis of GABAergic system. We discussed the potential value of the GABAergic system in treating depression, which may provide strategic suggestions for future research and more ideas for clinicians and researchers.

## Author Contributions

JL and KL conceived and designed the study. PH and MC collected data based on WOS. JL, MS, and FL analyzed by using CiteSpace. KL and WW reviewed and revised the manuscript. All authors contributed to the article and approved the submitted version.

## Funding

This work was supported by Guangdong Basic and Applied Basic Research Foundation 2018A0303130259 (Regulation of GABAA-mediated tonic inhibition on cognitive impairment in Parkinson's disease and its mechanism) and by Guangdong Basic and Applied Basic Research Foundation 2021A1515012481 (Role of projection of cholinergic neurons from medial septal nucleus to hippocampus in re-consolidation of cocaine reward memory).

## Conflict of Interest

WW was employed by Guangzhou Southern Medical Laboratory Animal Sci. and Tech. Co., Ltd. The remaining authors declare that the research was conducted in the absence of any commercial or financial relationships that could be construed as a potential conflict of interest.

## Publisher's Note

All claims expressed in this article are solely those of the authors and do not necessarily represent those of their affiliated organizations, or those of the publisher, the editors and the reviewers. Any product that may be evaluated in this article, or claim that may be made by its manufacturer, is not guaranteed or endorsed by the publisher.

## References

[1] Ledford H. Medical research: if depression were cancer. Nature. (2014) 515:182–4. 10.1038/515182a 25391943

[2] WHO. Depression and Other Common Mental Disorders: Global Health Estimates. Geneva, Switzerland: World Health Organization (2017).

[3] Smith K. Mental health: a world of depression. Nature. (2014) 515:181. 10.1038/515180a 25391942

[4] Kendler KS. The phenomenology of major depression and the representativeness and nature of DSM criteria. Am J Psychiatry. (2016) 173:771–80. 10.1176/appi.ajp.2016.15121509 27138588

[5] Tatsumi M Groshan K Blakely RD Richelson E. Pharmacological profile of antidepressants and related compounds at human monoamine transporters. Eur J Pharmacol. (1997) 340:249–58. 10.1016/S0014-2999(97)01393-9 9537821

[6] Glowinski J Axelrod J. Inhibition of uptake of tritiated-noradrenaline in the intact rat brain by imipramine and structurally related compounds. Nature. (1964) 204:1318–9. 10.1038/2041318a0 14254430

[7] Ross SB Renyi AL. Inhibition of the uptake of tritiated 5-hydroxytryptamine in brain tissue. Eur J Pharmacol. (1969) 7:270–7. 10.1016/0014-2999(69)90091-0 5351984

[8] Bauer M Bschor T Pfennig A Whybrow PC Angst J Versiani M. World Federation of Societies of Biological Psychiatry (WFSBP) guidelines for biological treatment of unipolar depressive disorders in primary care. World J Biol Psychiatry. (2007) 8:67–104. 10.1080/15622970701227829 17455102

[9] Luscher B Shen Q Sahir N. The GABAergic deficit hypothesis of major depressive disorder. Mol Psychiatry. (2011) 16:383–406. 10.1038/mp.2010.120 21079608 PMC3412149

[10] Vollenweider I Smith KS Keist R Rudolph U. Antidepressant-like properties of α2-containing GABA(A) receptors. Behav Brain Res. (2011) 217:77–80. 10.1016/j.bbr.2010.10.009 20965216 PMC3023881

[11] Gold PW Chrousos GP. Organization of the stress system and its dysregulation in melancholic and atypical depression: high vs. low CRH/NE states. Mol Psychiatry. (2002) 7:254–75. 10.1038/sj.mp.4001032 11920153

[12] MacKenzie G Maguire J. Chronic stress shifts the GABA reversal potential in the hippocampus and increases seizure susceptibility. Epilepsy Res. (2015) 109:13–27. 10.1016/j.eplepsyres.2014.10.003 25524838 PMC4272449

[13] Allen MJ Sabir S Sharma S. GABA receptor. In: StatPearls. Treasure Island, FL: StatPearls Publishing (2021). 30252380

[14] Jewett BE Sharma S. Physiology, GABA. In: StatPearls. Treasure Island, FL: StatPearls Publishing (2021).

[15] Fogaça MV Duman RS. Cortical GABAergic dysfunction in stress and depression: new insights for therapeutic interventions. Front Cell Neurosci. (2019) 13:87. 10.3389/fncel.2019.00087 30914923 PMC6422907

[16] Sarasa SB Mahendran R Muthusamy G Thankappan B Selta DRF Angayarkanni J. Brief review on the non-protein amino acid, gamma-amino butyric acid (GABA): its production and role in microbes. Curr Microbiol. (2020) 77:534–44. 10.1007/s00284-019-01839-w 31844936

[17] Petty F Sherman AD. Plasma GABA levels in psychiatric illness. J Affect Disord. (1984) 6:131–8. 10.1016/0165-0327(84)90018-1 6233345

[18] Gold BI Bowers MB Jr Roth RH Sweeney DW GABA. levels in CSF of patients with psychiatric disorders. Am J Psychiatry. (1980) 137:362–4. 10.1176/ajp.137.3.362 7356067

[19] Gerner RH Hare TA CSF GABA. In normal subjects and patients with depression, schizophrenia, mania, and anorexia nervosa. Am J Psychiatry. (1981) 138:1098–101. 10.1176/ajp.138.8.1098 7258390

[20] Hasler G van der Veen JW Tumonis T Meyers N Shen J Drevets WC. Reduced prefrontal glutamate/glutamine and gamma-aminobutyric acid levels in major depression determined using proton magnetic resonance spectroscopy. Arch Gen Psychiatry. (2007) 64:193–200. 10.1001/archpsyc.64.2.193 17283286

[21] Gabbay V Mao X Klein RG Ely BA Babb JS Panzer AM. Anterior cingulate cortex γ-aminobutyric acid in depressed adolescents: relationship to anhedonia. Arch Gen Psychiatry. (2012) 69:139–49. 10.1001/archgenpsychiatry.2011.131 21969419 PMC3711232

[22] Song Z Huang P Qiu L Wu Q Gong Q Zhang B. Decreased occipital GABA concentrations in patients with first-episode major depressive disorder: a magnetic resonance spectroscopy study. Sheng Wu Yi Xue Gong Cheng Xue Za Zhi. (2012) 29:233–6. (in Chinese). 22616164

[23] Schür RR Draisma LW Wijnen JP Boks MP Koevoets MG Joëls M. Brain GABA levels across psychiatric disorders: a systematic literature review and meta-analysis of (1) H-MRS studies. Hum Brain Mapp. (2016) 37:3337–52. 10.1002/hbm.23244 27145016 PMC6867515

[24] Godfrey KEM Gardner AC Kwon S Chea W Muthukumaraswamy SD. Differences in excitatory and inhibitory neurotransmitter levels between depressed patients and healthy controls: a systematic review and meta-analysis. J Psychiatr Res. (2018) 105:33–44. 10.1016/j.jpsychires.2018.08.015 30144668

[25] Khisti RT Chopde CT Jain SP. Antidepressant-like effect of the neurosteroid 3alpha-hydroxy-5alpha-pregnan-20-one in mice forced swim test. Pharmacol Biochem Behav. (2000) 67:137–43. 10.1016/S0091-3057(00)00300-2 11113493

[26] Pehrson AL Sanchez C. Altered γ-aminobutyric acid neurotransmission in major depressive disorder: a critical review of the supporting evidence and the influence of serotonergic antidepressants. Drug Des Devel Ther. (2015) 9:603–24. 10.2147/DDDT.S62912 25653499 PMC4307650

[27] Croarkin PE MacMaster FP. Transcranial magnetic stimulation for adolescent depression. Child Adolesc Psychiatr Clin N Am. (2019) 28:33–43. 10.1016/j.chc.2018.07.003 30389074 PMC6221455

[28] Chen C. Searching for intellectual turning points: progressive knowledge domain visualization. Proc Natl Acad Sci USA. (2004) 101(suppl):5303–10. 10.1073/pnas.0307513100 14724295 PMC387312

[29] Chen C. CiteSpace II: detecting and visualizing emerging trends and transient patterns in scientific literature. New York, NY: Wiley (2006).

[30] Yang J Cheng C Shen S Yang S. Comparison of complex network analysis software: Citespace, SCI2 and Gephi. In: IEEE International Conference on Big Data Analysis. (2017) 2017:169–72. 10.1109/ICBDA.2017.8078800

[31] Tremblay R Lee S Rudy B. GABAergic interneurons in the neocortex: from cellular properties to circuits. Neuron. (2016) 91:260–92. 10.1016/j.neuron.2016.06.033 27477017 PMC4980915

[32] WHO. World Misses Most 2020 Mental Health Targets; Extension of WHO Mental Health Action Plan to 2030 Provides New Opportunity for Progress. Geneva: World Health Organization (2021).

[33] Mazza MG De Lorenzo R Conte C Poletti S Vai B Bollettini I. Anxiety and depression in COVID-19 survivors: role of inflammatory and clinical predictors. Brain Behav Immun. (2020) 89:594–600. 10.1016/j.bbi.2020.07.037 32738287 PMC7390748

[34] Abdel-Bakky MS Amin E Faris TM Abdellatif AAH. Mental depression: relation to different disease status, newer treatments and its association with COVID-19 pandemic (Review). Mol Med Rep. (2021) 24:839. 10.3892/mmr.2021.12479 34633054 PMC8524409

[35] Santomauro DF Herrera AM Shadid J Zheng P Ashbaugh C. COVID-19 Mental Disorders Collaborators. Global prevalence and burden of depressive and anxiety disorders in 204 countries and territories in 2020 due to the COVID-19 pandemic. Lancet. (2021) 398:1700–12. 10.1016/S0140-6736(21)02143-7 34634250 PMC8500697

[36] WHO. Wake-Up Call to All Countries to Step Up Mental Health Services and Support. Geneva: World Health Organization (2022).

[37] Magnúsdóttir I Lovik A Unnarsdóttir AB McCartney D Ask H Kõiv K. Acute COVID-19 severity and mental health morbidity trajectories in patient populations of six nations: an observational study. Lancet Public Health. (2022) 2022:S2468-2667(22)00042-1. 10.1016/S2468-2667(22)00042-1 35298894 PMC8920517

[38] Price RB Shungu DC Mao X Nestadt P Kelly C Collins KA. Amino acid neurotransmitters assessed by proton magnetic resonance spectroscopy: relationship to treatment resistance in major depressive disorder. Biol Psychiatry. (2009) 65:792–800. 10.1016/j.biopsych.2008.10.025 19058788 PMC2934870

[39] Gao SF Bao AM. Corticotropin-releasing hormone, glutamate, and γ-aminobutyric acid in depression. Neuroscientist. (2011) 17:124–44. 10.1177/1073858410361780 20236945

[40] Gao SF Klomp A Wu JL Swaab DF Bao AM. Reduced GAD(65/67) immunoreactivity in the hypothalamic paraventricular nucleus in depression: a postmortem study. J Affect Disord. (2013) 149:422–5. 10.1016/j.jad.2012.12.003 23312397

[41] Douillard-Guilloux G Lewis D Seney ML Sibille E. Decrease in somatostatin-positive cell density in the amygdala of females with major depression. Depress Anxiety. (2017) 34:68–78. 10.1002/da.22549 27557481 PMC5222785

[42] Lim B Sproule BA Zahra Z Sunderji N Kennedy SH Rizvi SJ. Understanding the effects of chronic benzodiazepine use in depression: a focus on neuropharmacology. Int Clin Psychopharmacol. (2020) 35:243–53. 10.1097/YIC.0000000000000316 32459725

[43] Geng C Guo Y Wang C Liao D Han W Zhang J. Systematic impacts of chronic unpredictable mild stress on metabolomics in rats. Sci Rep. (2020) 10:700. 10.1038/s41598-020-57566-x 31959868 PMC6971284

[44] Srivastava I Vazquez-Juarez E Henning L Gómez-Galán M Lindskog M. Blocking astrocytic GABA restores synaptic plasticity in prefrontal cortex of rat model of depression. Cells. (2020) 9:1705. 10.3390/cells9071705 32708718 PMC7408154

[45] Adell A. Brain NMDA Receptors in schizophrenia and depression. Biomolecules. (2020) 10:947. 10.3390/biom10060947 32585886 PMC7355879

[46] Gerhard DM Pothula S Liu RJ Wu M Li XY Girgenti MJ. GABA interneurons are the cellular trigger for ketamine's rapid antidepressant actions. J Clin Invest. (2020) 130:1336–49. 10.1172/JCI130808 31743111 PMC7269589

[47] Fava M. Diagnosis and definition of treatment-resistant depression. Biol Psychiatry. (2003) 53:649–59. 10.1016/S0006-3223(03)00231-2 12706951

[48] Gaynes BN Lux L Gartlehner G Asher G Forman-Hoffman V Green J. Defining treatment-resistant depression. Depress Anxiety. (2020) 37:134–145. 10.1002/da.22968 31638723

[49] Ma K Xu A Cui S Sun MR Xue YC Wang J. Impaired GABA synthesis, uptake and release are associated with depression-like behaviors induced by chronic mild stress. Transl Psychiatry. (2016) 6:e910. 10.1038/tp.2016.181 27701406 PMC5315548

[50] Czéh B Varga ZK Henningsen K Kovács GL Miseta A Wiborg O. Chronic stress reduces the number of GABAergic interneurons in the adult rat hippocampus, dorsal-ventral and region-specific differences. Hippocampus. (2015) 25:393–405. 10.1002/hipo.22382 25331166

[51] Banasr M Lepack A Fee C Duric V Maldonado-Aviles J DiLeone R. Characterization of GABAergic marker expression in the chronic unpredictable stress model of depression. Chronic Stress. (2017) 1:2470547017720459. 10.1177/2470547017720459 28835932 PMC5565173

[52] Fatemi SH Folsom TD GABA. receptor subunit distribution and FMRP-mGluR5 signaling abnormalities in the cerebellum of subjects with schizophrenia, mood disorders, and autism. Schizophr Res. (2015) 167:42–56. 10.1016/j.schres.2014.10.010 25432637 PMC5301472

[53] Liang M Zhong H Rong J Li Y Zhu C Zhou L. Postnatal lipopolysaccharide exposure impairs adult neurogenesis and causes depression-like behaviors through astrocytes activation triggering GABAA receptor downregulation. Neuroscience. (2019) 422:21–31. 10.1016/j.neuroscience.2019.10.025 31682957

[54] Olsen RW Sieghart W. GABAA receptors: subtypes provide diversity of function and pharmacology. Neuropharmacology. (2009) 56:141–8. 10.1016/j.neuropharm.2008.07.045 18760291 PMC3525320

[55] Klein RL Harris RA. Regulation of GABAA receptor structure and function by chronic drug treatments *in vivo* and with stably transfected cells. Jpn J Pharmacol. (1996) 70:1–15. 10.1254/jjp.70.1 8822084

[56] Hewitt SA Wamsteeker JI Kurz EU Bains JS. Altered chloride homeostasis removes synaptic inhibitory constraint of the stress axis. Nat Neurosci. (2009) 12:438–43. 10.1038/nn.2274 19252497

[57] Duman RS Sanacora G Krystal JH. Altered connectivity in depression: GABA and glutamate neurotransmitter deficits and reversal by novel treatments. Neuron. (2019) 102:75–90. 10.1016/j.neuron.2019.03.013 30946828 PMC6450409

[58] Hill DR Bowery NG. 3H-baclofen and 3H-GABA bind to bicuculline-insensitive GABA B sites in rat brain. Nature. (1981) 290:149–52. 10.1038/290149a0 6259535

[59] Olsen RW Sieghart W. International Union of Pharmacology. LXX subtypes of gamma-aminobutyric acid(A) receptors: classification on the basis of subunit composition, pharmacology, and function update. Pharmacol Rev. (2008) 60:243–60. 10.1124/pr.108.00505 18790874 PMC2847512

[60] Mannoury la Cour C Hanoun N Melfort M Hen R Lesch KP Hamon M Lanfumey L. GABA(B) receptors in 5-HT transporter- and 5-HT1A receptor-knock-out mice: further evidence of a transduction pathway shared with 5-HT1A receptors. J Neurochem. (2004) 89:886–96. 10.1111/j.1471-4159.2004.02367.x 15140188

[61] Cryan JF Leonard BE. 5-HT1A and beyond: the role of serotonin and its receptors in depression and the antidepressant response. Hum Psychopharmacol. (2000) 15:113–35. 10.1002/(SICI)1099-1077(200003)15:2<113::AID-HUP150>3.0.CO;2-W 12404340

[62] Shen Q Lal R Luellen BA Earnheart JC Andrews AM Luscher B. gamma-Aminobutyric acid-type A receptor deficits cause hypothalamic-pituitary-adrenal axis hyperactivity and antidepressant drug sensitivity reminiscent of melancholic forms of depression. Biol Psychiatry. (2010) 68:512–20. 10.1016/j.biopsych.2010.04.024 20579975 PMC2930197

[63] Rush AJ Trivedi MH Wisniewski SR Nierenberg AA Stewart JW Warden D. Acute and longer-term outcomes in depressed outpatients requiring one or several treatment steps: a STAR^*^D report. Am J Psychiatry. (2006) 163:1905–17. 10.1176/ajp.2006.163.11.1905 17074942

[64] Klomjai W Katz R Lackmy-Vallée A. Basic principles of transcranial magnetic stimulation (TMS) and repetitive TMS (rTMS). Ann Phys Rehabil Med. (2015) 58:208–13. 10.1016/j.rehab.2015.05.005 26319963

[65] Rachid F. Maintenance repetitive transcranial magnetic stimulation (rTMS) for relapse prevention in with depression: a review. Psychiatry Res. (2018) 262:363–72. 10.1016/j.psychres.2017.09.009 28951141

[66] Voineskos D Blumberger DM Rogasch NC Zomorrodi R Farzan F Foussias G. Neurophysiological effects of repetitive transcranial magnetic stimulation (rTMS) in treatment resistant depression. Clin Neurophysiol. (2021) 132:2306–16. 10.1016/j.clinph.2021.05.008 34167891

[67] Godfrey KEM Muthukumaraswamy SD Stinear CM Hoeh N. Effect of rTMS on GABA and glutamate levels in treatment-resistant depression: An MR spectroscopy study. Psychiatry Res Neuroimaging. (2021) 317:111377. 10.1016/j.pscychresns.2021.111377 34479176

[68] Scangos KW Khambhati AN Daly PM Makhoul GS Sugrue LP Zamanian H. Closed-loop neuromodulation in an individual with treatment-resistant depression. Nat Med. (2021) 27:1696–700. 10.1038/s41591-021-01480-w 34608328 PMC11219029

[69] Kaster TS Fitzgerald PB Downar J Vila-Rodriguez F Daskalakis ZJ Blumberger DM. Considerable evidence supports rTMS for treatment-resistant depression. J Affect Disord. (2020) 263:549–51. 10.1016/j.jad.2019.11.017 31727396

[70] Folkerts HW Michael N Tölle R Schonauer K Mücke S Schulze-Mönking H. Electroconvulsive therapy vs. paroxetine in treatment-resistant depression—a randomized study. Acta Psychiatr Scand. (1997) 96:334–42. 10.1111/j.1600-0447.1997.tb09926.x 9395150

[71] Chase HW Boudewyn MA Carter CS Phillips ML. Transcranial direct current stimulation: a roadmap for research, from mechanism of action to clinical implementation. Mol Psychiatry. (2020) 25:397–407. 10.1038/s41380-019-0499-9 31455860 PMC6981019

[72] Milev RV Giacobbe P Kennedy SH Blumberger DM Daskalakis ZJ Downar J. Canadian network for mood and anxiety treatments (CANMAT) 2016 clinical guidelines for the management of adults with major depressive disorder: section 4. Neurostimulation treatments. Can J Psychiatry. (2016) 61:561–75. 10.1177/0706743716660033 27486154 PMC4994792

[73] Johnson RL Wilson CG A. review of vagus nerve stimulation as a therapeutic intervention. J Inflamm Res. (2018) 11:203–13. 10.2147/JIR.S163248 29844694 PMC5961632

[74] Qiao H Li MX Xu C Chen HB An SC Ma XM. Dendritic spines in depression: what we learned from animal models. Neural Plast. (2016) 2016:8056370. 10.1155/2016/8056370 26881133 PMC4736982

[75] Cryan JF Holmes A. The ascent of mouse: advances in modelling human depression and anxiety. Nat Rev Drug Discov. (2005) 4:775–90. 10.1038/nrd1825 16138108

[76] Duman RS Aghajanian GK Sanacora G Krystal JH. Synaptic plasticity and depression: new insights from stress and rapid-acting antidepressants. Nat Med. (2016) 22:238–49. 10.1038/nm.4050 26937618 PMC5405628

[77] Möhler H. The GABA system in anxiety and depression and its therapeutic potential. Neuropharmacology. (2012) 62:42–53. 10.1016/j.neuropharm.2011.08.040 21889518

[78] Ionescu DF Rosenbaum JF Alpert JE. Pharmacological approaches to the challenge of treatment-resistant depression. Dialogues Clin Neurosci. (2015) 17:111–26. 10.31887/DCNS.2015.17.2/dionescu 26246787 PMC4518696

[79] Ngo DH Vo TS. An updated review on pharmaceutical properties of gamma-aminobutyric acid. Molecules. (2019) 24:2678. 10.3390/molecules24152678 31344785 PMC6696076

[80] Selye H. Anesthetic effect of steroid hormones. Proc Soc Exp Biol Med. (1941) 46:116–21. 10.3181/00379727-46-11907

[81] Selye H. The antagonism between anesthetic steroid hormones and pentamethylenetetrazol (metrazol). J Lab Clin Med. (1942).

[82] Harrison NL Simmonds MA. Modulation of the GABA receptor complex by a steroid anaesthetic. Brain Res. (1984) 323:287–92. 10.1016/0006-8993(84)90299-3 6098342

[83] Melcangi RC Panzica G Garcia-Segura LM. Neuroactive steroids: focus on human brain. Neuroscience. (2011) 191:1–5. 10.1016/j.neuroscience.2011.06.024 21704130

[84] Streeter CC Gerbarg PL Brown RP Scott TM Nielsen GH Owen L. Thalamic gamma aminobutyric acid level changes in major depressive disorder after a 12-week Iyengar yoga and coherent breathing intervention. J Altern Complement Med. (2020) 26:190–7. 10.1089/acm.2019.0234 31934793 PMC7074898

[85] Erchinger VJ Miller J Jones T Kessler U Bustillo J Haavik J. Anterior cingulate gamma-aminobutyric acid concentrations and electroconvulsive therapy. Brain Behav. (2020) 10:e01833. 10.1002/brb3.1833 32940003 PMC7667336

[86] Milak MS Rashid R Dong Z Kegeles LS Grunebaum MF Ogden RT. Assessment of relationship of ketamine dose with magnetic resonance spectroscopy of Glx and GABA responses in adults with major depression: a randomized clinical trial. JAMA Netw Open. (2020) 3:e2013211. 10.1001/jamanetworkopen.2020.13211 32785636 PMC7424409

[87] Semkovska M Quinlivan L O'Grady T Johnson R Collins A O'Connor J. Cognitive function following a major depressive episode: a systematic review and meta-analysis. Lancet Psychiatry. (2019) 6:851–61. 10.1016/S2215-0366(19)30291-3 31422920

[88] Prévot T Sibille E. Altered GABA-mediated information processing and cognitive dysfunctions in depression and other brain disorders. Mol Psychiatry. (2021) 26:151–67. 10.1038/s41380-020-0727-3 32346158

[89] Chisari M Eisenman LN Covey DF Mennerick S Zorumski CF. The sticky issue of neurosteroids and GABAA receptors. Trends Neurosci. (2010) 33:299–306. 10.1016/j.tins.2010.03.005 20409596 PMC2902671

[90] Frieder A Fersh M Hainline R Deligiannidis KM. Pharmacotherapy of postpartum depression: current approaches and novel drug development. CNS Drugs. (2019) 33:265–82. 10.1007/s40263-019-00605-7 30790145 PMC6424603

[91] Althaus AL Ackley MA Belfort GM Gee SM Dai J Nguyen DP. Preclinical characterization of zuranolone (SAGE-217), a selective neuroactive steroid GABAA receptor positive allosteric modulator. Neuropharmacology. (2020) 181:108333. 10.1016/j.neuropharm.2020.108333 32976892 PMC8265595

